# Autoimmune Inner Ear Disease from a Rheumatologic Perspective

**DOI:** 10.3390/diagnostics15131577

**Published:** 2025-06-21

**Authors:** Maximiliano Diaz-Menindez, Ana-Maria Chindris, Carolyn Mead-Harvey, Yan Li, Ronald R. Butendieck, Razvan M. Chirila, Katherine L. Britt, Florentina Berianu

**Affiliations:** 1 Division of General Internal Medicine, Mayo Clinic, Scottsdale, AZ 85259, USA; diazmenindez.maximiliano@mayo.edu; 2 Division of Endocrinology, Mayo Clinic, Jacksonville, FL 32224, USAklbritt@stetson.edu (K.L.B.); 3 Department of Biostatistics, Mayo Clinic, Scottsdale, AZ 85259, USA; meadharvey.carolyn@mayo.edu; 4 Division of Rheumatology, Mayo Clinic, Jacksonville, FL 32224, USAbutendieck.ronald@mayo.edu (R.R.B.J.); 5 Division of General Internal Medicine, Mayo Clinic, Jacksonville, FL 32224, USA; chirila.razvan@mayo.edu

**Keywords:** sensorineural hearing loss, autoimmune response, rheumatology, immunosuppression therapy

## Abstract

**Background/Objectives**: Autoimmune inner ear disease (AIED) causes sensorineural hearing loss that classically presents as fluctuating, asymmetric loss of hearing. Associated vestibular and other ear symptoms can be present in many patients. First-line treatment of AIED is high-dose corticosteroids. AIED can present either as a primary condition limited to ear involvement or secondary, as part of an underlying systemic autoimmune rheumatic disease, the most common of which include vasculitis and relapsing polychondritis. We described our cohort of primary AIED, including demographics, treatment, and outcomes. We excluded from this review sensorineural hearing loss in the context of vasculitis and relapsing polychondritis. **Methods**: We performed a chart review of patients with the diagnosis of AIED at Mayo Clinic and compared the cohort by sex. **Results**: Thirty-one patients met the inclusion criteria. The mean age was 48.5 years, and 17 were men. Patients were initially evaluated at the Department of Otorhinolaryngology or Internal Medicine, and 29 patients were subsequently referred to the Department of Rheumatology, with a mean of 12.2 weeks after the first evaluation. Treatment with corticosteroids showed improvement in hearing and vestibular symptoms during the first month but no further improvement by the end of the third month. Other immunosuppressive medications were used with various degrees of response. Methotrexate was the second most used therapy, with 11 of 17 patients reporting an improvement in symptoms. **Conclusions**: Corticosteroid therapy is an effective initial treatment for AIED and should be followed with corticosteroid-sparing agents to prevent further damage to the cochlea.

## 1. Introduction

Autoimmune inner ear disease (AIED) is a rare entity that presents with sensorineural hearing loss (SNHL). In the U.S., the incidence rate is less than 5 per 100,000 people [1]. The pathophysiology behind the disease is thought to be a humoral and cell-mediated process that damages the cochlea [2]. One study reported a higher prevalence of AIED in women 30 to 60 years of age [3], whereas another study noted a similar distribution between men and women [4]. Clinical presentation, as outlined by the 1994 National AIED Conference, includes a history of rapidly progressive, often fluctuating unilateral or bilateral sensorineural hearing loss, potentially associated with vestibular symptoms such as tinnitus, dizziness, and the presence of autoimmune serological markers [5]. It is important to know when symptoms that began as SNHL in AIED are progressive (weeks to months) rather than acute. The incidence of vestibular (e.g., dizziness, vertigo, and imbalance) and other (e.g., tinnitus, ear pressure, and aural fullness) ear symptoms has been reported to be as high as 50% [2,3]. Loveman et al. [4] reported that 30% of their patients presented with an associated systemic autoimmune disease (SAD). Although the antibodies against Heat-shock protein 70 have been identified in AIED patients with a sensitivity of 54% and specificity of 42% [6], the diagnosis of AIED remains challenging. Imaging such as magnetic resonance imaging is used to rule out anatomical causes of SNHL. An important indirect diagnostic criterion is represented by the initial response to corticosteroids, which suggests the disease’s immune-mediated nature. Corticosteroid-sparing agents have been used with various degrees of success [7,8,9]. When medical treatment is unsuccessful, resulting in severe bilateral SNHL, cochlear implants are an alternative therapy to restore hearing.

The aim of our study was to report the Mayo Clinic experience diagnosing and treating this rare condition.

## 2. Materials and Methods

### 2.1. Study Subjects, Data Collection, and Outcomes

This study was conducted in accordance with recognized ethical guidelines including the U.S. Common Rule and with the approval of our Institutional Review Board. We performed a retrospective chart review of patients diagnosed with AIED at all three Mayo Clinic locations (Jacksonville, FL, USA; Scottsdale, AZ, USA and Rochester MN, USA) between 1 January 2010 and 31 December 2021. We included patients initially seen in the Department of Rheumatology, Otorhinolaryngology/Audiology, or Internal Medicine. Inclusion criteria were patients with a documented diagnosis of AIED by an ENT, internist, or rheumatologist treated in our institution who had at least 3 months of follow-up.

Exclusion criteria included SNHL of other etiologies such as infections, tumors, anatomic abnormalities, and secondary AIED due to vasculitis (ANCA-associated vasculitis, Cogan syndrome, Kawasaki disease, and relapsing polychondritis).

Data collection included patient demographics (age at diagnosis, sex, and race), the first medical department visited, SNHL characteristics (type of onset, uni- or bilateral involvement, and progression), associated symptoms (vestibular, tinnitus, and aural fullness), predisposing factors, and response to therapy. The presence of other autoimmune systemic diseases at the time of diagnosis was also recorded. We collected audiometric evidence supporting sensorineural hearing loss (as an increase of 10 dB or greater in 2 or more frequencies or 15 dB or greater in 1 frequency). We recorded audiogram results at the time of diagnosis and 1 and 3 months after starting the treatment. The treatment efficacy was divided into two categories: subjective (patient-reported having a subjective improvement in SNHL) and objective (audiometric) improvement. Based on the available literature, we defined objective improvement as an increase of 10 dB or greater in two or more frequencies or 15 dB or greater in one frequency [10,11].

### 2.2. Statistical Analysis

Continuous variables were summarized using the sample mean (SD), median (IQR), and range. Categorical variables were summarized with numbers and percentages. Continuous variables were compared using Wilcoxon rank-sum tests; differences in categorical variables were tested using Fisher’s exact tests; differences in the onset period of symptoms were tested using a Cochran–Armitage trend test.

## 3. Results

Thirty-one patients met the inclusion criteria (Table 1). Seventeen patients (54.8%) were men, the mean (SD) age at diagnosis was 48.5 (16.0) years, and the majority were white. Women had an earlier age of diagnosis, with a mean of 43.1 years, compared to men, with a mean of 53 years of age. For most patients, initial evaluation was made in the Department of Otorhinolaryngology/Audiology (17, 54.8%). Twenty-five patients (80%) reported an onset of symptoms weeks or months prior to the first visit. Twenty-nine patients (93.5%) were referred to the Department of Rheumatology and were seen within a mean of 12.2 weeks from the initial evaluation. Tinnitus, aural fulness, and ear pressure were the three most commonly reported associated symptoms, followed by imbalance, vertigo, dizziness, and discharge (Table 1).

**Figure 1:**
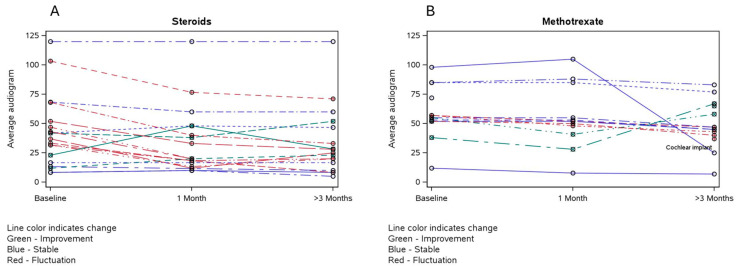
Response to treatment with corticosteroids (**A**) and methotrexate (**B**) in decibels from at 1 and 3 months. One patient in (**B**) who experienced a severe decline in hearing underwent a cochlear implant.

Treatment responses to corticosteroids, based on audiograms, are shown in Figure 1A and Table 2. Most patients initially treated with corticosteroids exhibited either improvement in symptoms or no progression, with the extent of response varying between each ear. However, after 3 months of corticosteroids, additional response was largely absent.

Patients treated with methotrexate experienced stability or further improvement, especially after 3 months of therapy (Figure 1B). Treatment with rituximab demonstrated improvement in both ears in one patient, whereas another patient experienced stability of SNHL in one ear but worsening in the other. Table 3 summarizes responses to therapy for the patients treated with other immunomodulators.

Table 4 shows patients’ perception of symptomatic improvement regarding SNHL with different treatments. Improvement was reported by 27 of 31 patients treated with prednisone, 11 of 17 treated with methotrexate, and all 9 patients who underwent intratympanic corticosteroid injection.

Specific treatment information for each of the 31 patients is presented in Table 5.

## 4. Discussion

Our study included 31 patients with primary AIED treated by a multidisciplinary team including audiologists, otolaryngologists, and rheumatologists. We found that most patients presented with associated tinnitus, aural fullness, ear pressure, and vestibular symptoms, which is a higher symptom burden than the 50% prevalence reported by Mijovic et al. [2]. However, Broughton et al. [10] reported that 79% of patients had vestibular and ear symptoms in their cohort. The mean age (48.5 years) and distribution of men and women (45.2 vs. 54.8) in our study were similar to those reported by Loveman et al. [4].

In our cohort, SNHL developed over weeks to months, with most symptoms reported as unilateral. In a study by McCabe et al. [12], 80% of patients who reported unilateral symptoms had bilateral involvement on audiograms. In our cohort, only 29% of patients with symptomatic unilateral SNHL had bilateral involvement on audiogram initially; however, progression to bilateral involvement was seen in 65% of our patients.

The diagnosis of AIED can be challenging, especially in patients who present without an associated SAD, and it should be differentiated from other causes of SNHL. Patient history helps in distinguishing from conditions such as Charcot–Maria–Tooth disease (hereditary), Lyme disease, toxoplasmosis, and exposure to ototoxic drugs (gentamicin, cisplatin). The clinical distinction between AIED and Meniere’s disease can be difficult, as Meniere’s has also been associated with systemic autoimmune conditions in some studies [13,14]. Although there is a significant overlap in symptomatology, Meniere’s presents primarily with vestibular symptoms, and the main histopathological feature is an excessive buildup of endolymph fluid in the membranous labyrinth of the inner ear (endolymphatic hydrops) [15].

Previous studies reported that 16–30% of patients with AIED have a systemic autoimmune disease at presentation [2,16]. After excluding the cases of SNHL in context of vasculitis or relapsing polychondritis, we found that seven (22.5%) patients in our cohort had an associated SAD: polymyalgia rheumatica (2), seronegative inflammatory arthritis (1), scleroderma with polymyositis (1), polymyalgia rheumatica (1), rheumatoid arthritis (1), and psoriatic arthritis (1) were seen; the rest of the patients did not present with a rheumatologic condition. It is likely that many patients who present initially with primary AIED later develop clinical features of an associated autoimmune systemic disease.

Treatment of AIED remains challenging. Optimal management requires a multidisciplinary team to provide a comprehensive approach. Mainstay therapy for AIED is oral corticosteroids, with an initial dose of prednisone up to 1 mg/kg, tapered over several weeks to months [7,10]. Prompt administration may result in the preservation of hearing in up to 60% of patients [17]. In our patient cohort, the treatment with corticosteroids was associated with notable improvement, based on audiograms, by the end of the first month of therapy, consistent with previously published studies [16].

However, similar to other inflammatory diseases, some patients experienced worsening symptoms either once corticosteroids were discontinued due to concern regarding adverse effects associated with long-term use [2,7] or due to a lack of response to therapy. For these patients, non-steroid immunosuppressive therapies were used, with variable results. Seventeen patients in our cohort received methotrexate, with symptom improvement in eleven. In an open-label study by Matteson et al., 9 of 17 patients with AIED treated with methotrexate reported 65% objective (audiometric data) improvement in symptoms and 35% subjective (patient-reported) improvement [18]. Comparatively, our study showed more subjective improvement with the use of methotrexate (64.7%). Due to the small cohorts and retrospective nature, the results of these studies should be interpreted with caution; prospective studies of longer duration are needed to better understand alternative treatments to non-steroid-sparing agents.

The results from our study indicate that initial treatment with corticosteroids followed by corticosteroid-sparing agents such as methotrexate may provide longer-lasting improvement in patients with AIED. Patients treated with corticosteroids exhibited improvement after 1 month of treatment but little to no improvement after 3 months. On the other hand, in patients taking methotrexate, improvement was noted on audiogram after 3 months of treatment compared to 1 month. This suggests that corticosteroids may be useful as bridge therapy until a corticosteroid-sparing medication such as methotrexate becomes effective. Our cohort also showed subjective improvement in symptoms with other immunosuppressive therapies. Improvement was reported by all three patients treated with azathioprine, two of three treated with adalimumab, and one of two treated with rituximab. In a prospective cohort of 20 patients, a 1-year course of azathyoprine added to the initial standard prednisone taper was associated with maintenance of the hearing threshold and a decrease in the rate of relapse [8].

The IL-1 receptor agonist anakinra was used in an open-label phase I/II clinical trial, supported by data indicating an increased expression of IL-1β in a subset of steroid-resistant individuals [19], with seven out of ten patients demonstrating audiometric response at the end of the trial [20]. None of the patients in our group received this type of therapy.

A retrospective study by Garcia-Berrocal et al. [21], reported that, of the 11 patients receiving intratympanic corticosteroid injections, 54% reported improvement in their symptoms. While all nine patients in our cohort receiving this treatment reported improvement initially, they noticed a decline in their hearing after a few months.

For patients with significant hearing loss refractory to immunosuppressive treatment, cochlear implants have been demonstrated to be a viable method of hearing rehabilitation [22]. In our cohort, one of the patients who experienced progressive hearing loss while treated with methotrexate underwent a cochlear implant procedure by the end of our review.

## 5. Conclusions

Despite the inherent limitations related to the small patient cohort and retrospective nature, this study highlights the complex clinical presentation and management of AIED within a multidisciplinary care model. Patients typically presented with a combination of auditory and vestibular symptoms, with tinnitus, aural fullness, and ear pressure being the most commonly reported. While corticosteroids remain the mainstay of initial therapy, our data suggest that their benefit diminishes after the first month, underscoring the need for a timely transition to steroid-sparing agents.

Methotrexate showed promise as a second-line treatment, with subjective and objective improvements noted particularly after three months of therapy. Other immunosuppressive agents, including azathioprine, adalimumab, and rituximab, also yielded symptomatic improvement in select patients, though responses were variable and often ear-specific.

Overall, our findings support a stepwise treatment approach that begins with corticosteroids and transitions to immunosuppressive therapy to sustain clinical benefit. However, variability in patient response and the retrospective design of the study highlight the need for prospective, controlled trials to better define treatment algorithms and long-term outcomes for AIED.

## Figures and Tables

**Table 1:** Autoimmune inner ear disease symptoms and course by gender.

	Gender			
	Male (*N* = 17)	Female (*N* = 14)	Total (*N* = 31)	*p*-Value
Age at diagnosis				0.095 ^1^
Mean (SD)	53.0 (17.05)	43.1 (13.26)	48.5 (16.02)	
Median (IQR)	54.0 (38.0, 66.0)	43.0 (36.0, 48.0)	47.0 (37.0, 63.0)	
Range	26.0, 78.0	23.0, 70.0	23.0, 78.0	
Race, *n* (%)				>0.999 ^2^
White	16 (94.1%)	13 (92.9%)	29 (93.5%)	
African American	1 (5.9%)	1 (7.1%)	2 (6.5%)	
First Consultation, *n* (%)				0.719 ^2^
ENT	8 (47.1%)	9 (64.3%)	17 (54.8%)	
Internal Medicine	9 (52.9%)	5 (35.7%)	14 (38.7%)	
Onset of symptoms, *n* (%)				>0.999 ^3^
Days	4 (23.5%)	2 (14.3%)	6 (19.4%)	
Weeks	9 (52.9%)	10 (71.4%)	19 (61.3%)	
Months	4 (23.5%)	2 (14.3%)	6 (19.4%)	
Symptom start, *n* (%)				>0.999 ^2^
Unilateral	11 (64.7%)	10 (71.4%)	21 (67.7%)	
Bilateral	6 (35.3%)	4 (28.6%)	10 (32.3%)	
Symptom progression, *n* (%)				>0.999 ^2^
Unilateral	4 (26.7%)	4 (30.8%)	8 (28.6%)	
Bilateral	11 (73.3%)	9 (69.2%)	20 (71.4%)	
Missing	2	1	3	
Tinnitus, *n* (%)	11 (64.7%)	9 (64.3%)	20 (64.5%)	>0.999 ^2^
Aural fullness, *n* (%)	7 (41.2%)	5 (35.7%)	12 (38.7%)	>0.999 ^2^
Ear pressure, *n* (%)	7 (41.2%)	5 (35.7%)	12 (38.7%)	>0.999 ^2^
Imbalance, *n* (%)	5 (29.4%)	3 (21.4%)	8 (25.8%)	0.698 ^2^
Vertigo, *n* (%)	2 (11.8%)	3 (21.4%)	5 (16.1%)	0.636 ^2^
Dizziness, *n* (%)	2 (11.8%)	3 (21.4%)	5 (16.1%)	0.636 ^2^
No symptoms, *n* (%)	1 (5.9%)	2 (14.3%)	3 (9.7%)	0.576 ^2^

^1^ Wilcoxon rank-sum test, *p*-value; ^2^ Fisher’s exact test, *p*-value; ^3^ Cochran–Armitage trend test.

**Table 2:** Treatments and outcomes based on audiogram results.

Treatment	Corticosteroids (*n* = 10)	Methotrexate (*n* = 8)
1 month of treatment		
Stable	40%	56.25%
Improvement	45%	12.5%
Fluctuation	10%	6.25%
No data	5%	25%
3 months of treatment		
Stable	65%	43.75%
Improvement	10%	25%
Fluctuation	10%	18.75%
No data	15%	12.5%

**Table 3:** Outcomes based on audiogram in individual patients receiving non-steroid treatment.

Patient	Treatment	Right Ear	Left Ear
12	Rituximab and azathioprine	Stable	Worsening
15	Rituximab	Improvement	Improvement
18	Mycophenolic acid	Improvement	Improvement
20	Azathioprine	Stable	Stable
24	Adalimumab	Improvement	Fluctuation
31	Azathioprine	Improvement	Fluctuation

**Table 4:** Treatments and outcomes based on subjective responses.

Treatment	Improvement, No. (%)	No Improvement, No. (%)
Prednisone (*n* = 31)	27 (87.1)	4 (12.9)
Intratympanic corticosteroid injection (*n* = 9)	9 (100.0)	0 (0.0)
Methotrexate (*n* = 17)	11 (64.7)	6 (35.3)
Azathioprine (*n* = 3)	3 (100.0)	0 (0.0)
Adalimumab (*n* = 3)	2 (66.7)	1 (33.3)
Rituximab (*n* = 2)	1 (50.0)	1 (50.0)
Mycophenolic acid (*n* = 1)	1 (100.0)	0 (0.0)
Etanercept (*n* = 1)	0 (0.0)	1 (100.0)

**Table 5:** Individual patient cases with demographics and treatments.

	Gender	Race	Onset of Symptoms	Age at Diagnosis	Initial Symptoms	First Audiogram	Vestibular Symptoms	Corticosteroid-Sparing Agents Used
1	Male	White	Months	70	Unilateral	Unilateral	Tinnitus, aural fullness, ear pressure	Methotrexate
2	Female	White	Months	23	Unilateral	Unilateral	Tinnitus, aural fullness, ear pressure	None
3	Male	White	Months	26	Unilateral	Bilateral	Imbalance	Methotrexate
4	Male	Black	Weeks	28	Bilateral	Bilateral	Imbalance, aural fullness, ear pressure	None
5	Female	White	Weeks	31	Unilateral	Bilateral	Tinnitus, aural fullness, ear pressure, vertigo, imbalance	Methotrexate
6	Female	White	Weeks	24	Unilateral	Bilateral	Dizziness, aural fullness, ear pressure	Methotrexate
7	Female	White	Weeks	37	Bilateral	Unilateral	Tinnitus, imbalance	Methotrexate
8	Male	White	Weeks	38	Unilateral	Unilateral	None	Methotrexate, azathioprine
9	Male	White	Weeks	37	Unilateral	Bilateral	Vertigo, tinnitus	None
10	Male	White	Months	39	Unilateral	Bilateral	Tinnitus, imbalance	Methotrexate; Etanercept; Adalimumab
11	Female	White	Weeks	36	Unilateral	Bilateral	Tinnitus, aural fullness, ear pressure, imbalance	Methotrexate; Adalimumab
12	Female	White	Weeks	42	Unilateral	Unilateral	None	Azathioprine; Rituximab
13	Female	White	Weeks	45	Unilateral	Bilateral	Tinnitus	Rituximab
14	Female	White	Weeks	47	Unilateral	Unilateral	Tinnitus, aural fullness, ear pressure	None
15	Female	Black	Weeks	44	Unilateral	Unilateral	Tinnitus	Mycophenolic acid
16	Female	White	Months	49	Bilateral	Bilateral	Vertigo, tinnitus	Azathioprine
17	Male	White	Weeks	53	Bilateral	Bilateral	Tinnitus, aural fullness, ear pressure, imbalance, dizziness	None
18	Male	White	Days	54	Bilateral	Bilateral	Tinnitus, aural fullness, ear pressure	Methotrexate
19	Female	White	Weeks	48	Bilateral	Bilateral	Tinnitus, dizziness	Methotrexate
20	Male	White	Weeks	54	Unilateral	Bilateral	Vertigo, dizziness	Methotrexate; Adalimumab
21	Male	White	Days	57	Unilateral	Unilateral	Tinnitus, aural fullness, ear pressure, imbalance	None
22	Male	White	Weeks	59	Unilateral	Bilateral	Tinnitus	Methotrexate
23	Female	White	Weeks	42	Unilateral	Bilateral	None	Methotrexate
24	Male	White	Weeks	66	Bilateral	Bilateral	Aural fullness, ear pressure	None
25	Female	White	Days	65	Bilateral	Bilateral	Vertigo	None
26	Female	White	Days	70	Unilateral	Bilateral	Dizziness	Methotrexate; Azathioprine
27	Male	White	Days	71	Unilateral	Unilateral	Tinnitus	Methotrexate
28	Male	White	Months	77	Bilateral	Bilateral	Aural fullness, ear pressure	Methotrexate
29	Male	White	Days	78	Unilateral	Unilateral	Tinnitus	None
30	Male	White	Weeks	63	Bilateral	Bilateral	Tinnitus	Methotrexate
31	Male	White	Weeks	31	Unilateral	Unilateral	Tinnitus	None

## Data Availability

The data that support the findings of this study are available upon request from the corresponding author. The data are not publicly available due to privacy or ethical restrictions.
